# Optimization of the Preparation Process of Glucuronomannan Oligosaccharides and Their Effects on the Gut Microbiota in MPTP-Induced PD Model Mice

**DOI:** 10.3390/md22050193

**Published:** 2024-04-25

**Authors:** Baoxiang Wang, Lihua Geng, Jing Wang, Yuxi Wei, Changhui Yan, Ning Wu, Yang Yue, Quanbin Zhang

**Affiliations:** 1 College of Life Sciences, Qingdao University, 308 Ningxia Road, Qingdao 266003, China; wangbaoxiangcn@163.com (B.W.); yuxiw729@163.com (Y.W.); 2 CAS and Shandong Province Key Laboratory of Experimental Marine Biology, Institute of Oceanology, Chinese Academy of Sciences, Qingdao 266071, China; jingwang@qdio.ac.cn (J.W.); ych2747141850@163.com (C.Y.); wuning@qdio.ac.cn (N.W.); yueyang@qdio.ac.cn (Y.Y.); 3 Laboratory for Marine Biology and Biotechnology, Qingdao Marine Science and Technology Center, Qingdao 266237, China; 4 Center for Ocean Mega-Science, Chinese Academy of Sciences, Qingdao 266071, China; 5 Key Laboratory of Optic-Electric Sensing and Analytical Chemistry for Life Science, MOE, College of Chemistry and Molecular Engineering, Qingdao University of Science and Technology, Qingdao 266042, China

**Keywords:** glucuronomannan, oligosaccharide, Parkinson’s disease (PD), gut microbiota

## Abstract

Parkinson’s disease (PD) is a prevalent neurodegenerative disorder, and accumulating evidence suggests a link between dysbiosis of the gut microbiota and the onset and progression of PD. In our previous investigations, we discovered that intraperitoneal administration of glucuronomannan oligosaccharides (GMn) derived from *Saccharina japonica* exhibited neuroprotective effects in a 1-methyl-4-phenyl-1,2,3,6-tetrahydropyridine (MPTP)-induced PD mouse model. However, the complicated preparation process, difficulties in isolation, and remarkably low yield have constrained further exploration of GMn. In this study, we optimized the degradation conditions in the preparation process of GMn through orthogonal experiments. Subsequently, an MPTP-induced PD model was established, followed by oral administration of GMn. Through a stepwise optimization, we successfully increased the yield of GMn, separated from crude fucoidan, from 1~2/10,000 to 4~8/1000 and indicated the effects on the amelioration of MPTP-induced motor deficits, preservation of dopamine neurons, and elevation in striatal neurotransmitter levels. Importantly, GMn mitigated gut microbiota dysbiosis induced by MPTP in mice. In particular, GM2 significantly reduced the levels of *Akkermansia*, Verrucomicrobiota, and *Lactobacillus*, while promoting the abundance of *Roseburia* and *Prevotella* compared to the model group. These findings suggest that GM2 can potentially suppress PD by modulating the gut microbiota, providing a foundation for the development of a novel and effective anti-PD marine drug.

## 1. Introduction

Parkinson’s disease (PD) ranks as the second most prevalent neurodegenerative disorder, following Alzheimer’s disease (AD). It is characterized by the degeneration of dopaminergic neurons in the substantia nigra pars compacta (SNpc) and the abnormal deposition of α-synuclein in the Lewy bodies, and a high rate of disability and mortality associated with the disease [1]. Currently, PD affects 1% of the global population aged 65 and above, with an escalating incidence due to the aging demographic [2]. Currently, the primary drugs used to treat PD include catecholamine-O-methyltransferase inhibitors, anticholinergic drugs, dopamine agonists, levodopa, and monoamine oxidase inhibitors [3]. Among these, levodopa is the most efficacious for addressing movement disorders in PD. However, its efficacy diminishes after long-term use, and it can cause fatal complications and side effects. Therefore, there is an urgent need to develop a long-acting, safe anti-Parkinson’s drug without side effects.

Natural molecules derived from plants and other sources have been found to have significant ameliorative effects in chronic diseases and are considered safe and effective alternatives to current synthetic drugs [4]. Research has demonstrated that bioactive polysaccharides or oligosaccharides derived from animals, plants, algae, and fungi possess the potential to prevent or treat neurodegenerative diseases [5,6,7,8,9]. Fucoidan, a sulfated polysaccharide rich in L-fucose, exhibits diverse biological activities and holds promise as a therapeutic agent for neurodegenerative diseases [10,11]. Our previous studies have elucidated the anti-PD role of fucoidan and its sulfated hetero-polysaccharides [12,13,14]. In recent years, we reported GMn isolated from fucoidan, with their comprehensive structural characterization [15]. Additionally, preliminary investigations into the anti-PD mechanisms of GM2 were conducted [16]. The findings indicated that GM2 could prevent MPP+-induced apoptosis in PC12 cells and the loss of dopamine neurons in PD mice by enhancing mitochondrial function and cellular autophagy [17]. It is worth noting that previous studies on the anti-PD effects of GMn in Parkinsonism mice used injectable administration due to yield limitations. However, PD is a chronic neurodegenerative disease that requires long-term medication, and oral administration stands out as a more convenient and prevalent method of delivery. Whether oral administration of GMn to PD animals have equally neuroprotective effect needs further investigation.

In addition to exhibiting motor symptoms such as bradykinesia, muscle tonus, resting tremor, and postural and gait disturbances, patients with PD also exhibit non-motor symptoms including gastrointestinal dysfunction, olfactory dysfunction, cognitive deficits, psychiatric symptoms, sleep disturbances, autonomic dysfunction, pain, and fatigue [1,2]. Increasing evidence suggests the existence of a microbiota–gut–brain axis, thereby contributing to the development of motor or non-motor symptoms in PD [18,19]. Dysbiosis of the gut microbiota may initiate or exacerbate the progression of PD through various mechanisms, including increasing intestinal permeability, exacerbating neuroinflammation, aggregating abnormal levels of α-synuclein fibrils, enhancing oxidative stress, and reducing neurotransmitter production [20,21]. Consequently, the gut microbiota is considered a promising diagnostic and therapeutic target for PD [22]. Therefore, this study aims to optimize the existing preparation process of GMn by employing orthogonal experiments to explore efficient degradation conditions, and verify the potential anti-PD effects of GM1 and GM2. Additionally, we intend to elucidate the role of GM1 and GM2 on gut microbiota and their potential regulatory mechanisms for the imbalance of gut microbial in MPTP-induced PD mice.

## 2. Results

### 2.1. Optimizing the Preparation Process and Preparing GMn

**Figure 1:**
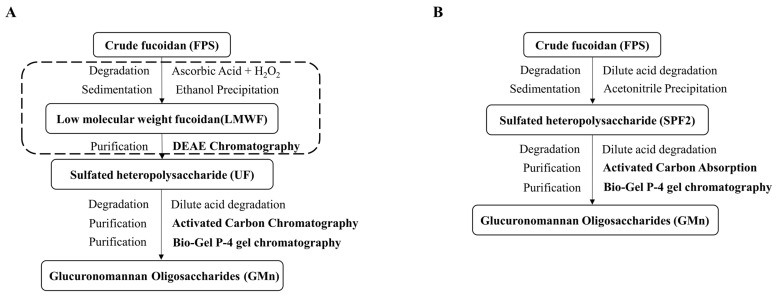
The flow chart for preparing GMn. (**A**) The flow chart of the previous process [15]. (**B**) The flow chart of the optimized process.

**Figure 2:**
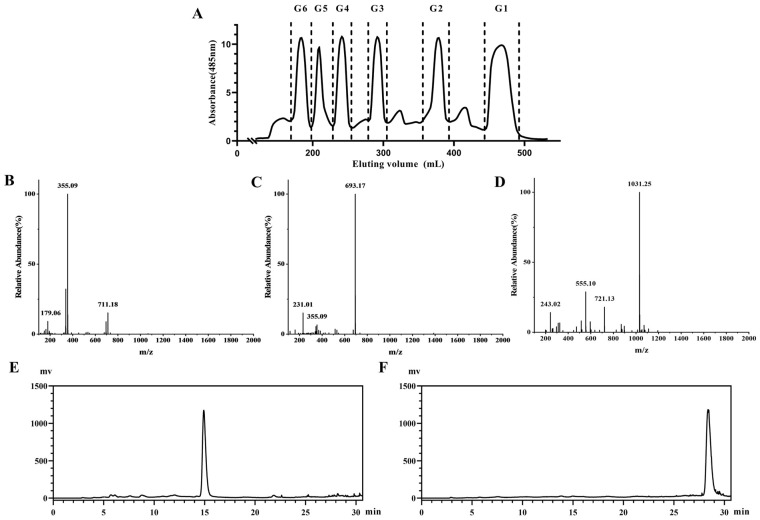
Preparation and characterization of GMn. (**A**) Gel filtration chromatography of Y2 on a Bio-Gel P-4 gel column. (**B**) Negative ion mode ESI-MS spectra of G2 (GM1, [M−H]^−^ = 355.09 *m*/z) (**C**) Negative ion mode ESI-MS spectra of G3 (GM2, [M−H]^−^ = 693.17 *m*/z). (**D**) Negative ion mode ESI-MS spectra of G4 (GM3, [M−H]^−^ = 1031.25 *m*/z). (**E**) HPLC spectrum of G2 (GM1). (**F**) HPLC spectrum of G3 (GM2).

GMn were prepared from the hydrolyzed sulfated polysaccharide. In order to improve their yield, this study utilized crude fucoidan extracted from *S. japonica* for a graded purification after a two-step degradation process. This approach eliminated the need for multistep chromatography, improved sample yield, and simplified the process flow. SPF2 with a high content of glucuronic acid and mannose was used to optimize the degradation process. However, acid degradation emerged as a pivotal control step in GMn production. The degradation temperature (A) and degradation time (B) of SPF2 were optimized using a two-factor, three-level orthogonal test. After degradation, GMn were purified according to the methods described in Figure 1B. Table 1 indicated that the relative abundance of GM1 in the degraded products ranged from 1.27% to 17.36%, while GM2 ranged from 0.89% to 13.44%. Table 2 further analyzed the orthogonal results, revealing the effects of degradation conditions on the relative abundance of GM1 and GM2 in the following descending order: A > B. Consequently, the crucial factor in the GMn preparation process was the degradation temperature. The optimal condition was A3B1, which specified the ideal degradation conditions of SPF2 for GMn preparation were 4% sulfuric acid at 100 °C, for 3.5 h. The ANOVA revealed that only temperature had a significant effect (*p* < 0.05) on GM2 in the degradation product, which also had a notable effect on GM1 (Table 3). After degradation under these conditions, adsorption was performed using activated carbon. The 50% ethanol-eluted fraction (Y2) was purified further through using a Bio-Gel P-4 gel column to yield six components. (Figure 2A). The negative ion mode ESI-MS analysis revealed that the G2, G3, and G4 fractions corresponded to glucuronomannan disaccharide (GM1), glucuronomannan tetrasaccharide (GM2), and glucuronomannan hexasaccharide (GM3), respectively (Figure 2B–D). The purity and structure of GM1and GM2 remained consistent with previous preparations (Figure 2E,F and Figure S1) [15,23], while the yield significantly improved. In conclusion, employing fucoidan as the raw material and subjecting it to degradation and purification under the optimized conditions, we achieved a yield of 8.173 ± 0.731‰ for GM1, 4.875 ± 0.379‰ for GM2, and 3.945 ± 0.377‰ for GM3.

### 2.2. Protective Effects of GMn on 6-OHDA-Induced Neurotoxicity on SH-SY5Y Cells

**Figure 3:**
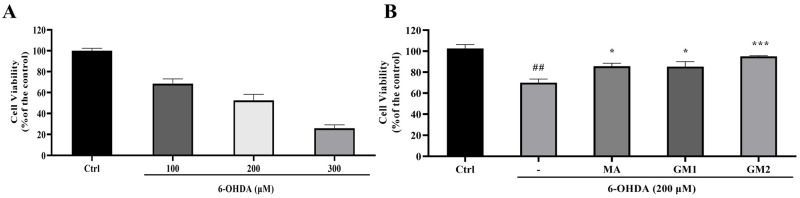
Neuroprotective activities of GMn on SH-SY5Y cells. (**A**) Cytotoxic effect of different concentrations of 6-OHDA in SH-SY5Y cells. (**B**) Effect of GM1 and GM2 pretreatment on SH-SY5Y cell viability after 6-OHDA treatment. Ctrl: the cell treated with PBS; MA: the cell treated with Madopar (100 mM), used as a positive control; GM1/2: the cell treated with GM1 or GM2 (200 μg/mL). The values are given as mean ± SEM. ^##^ *p* < 0.01 versus the control cells; * *p* < 0.05 or *** *p* < 0.001 versus the cells treated with 6-OHDA alone.

6-OHDA is commonly employed as inducer for in vitro neuronal cell injury models [24,25]. Thus, in this study, it was used to validate the potential neuroprotective effects of GMn in vitro. The study found that cell viability was significantly reduced by 6-OHDA treatment in a concentration-dependent manner (Figure 3A). Cell viability decreased to 52.50 ± 5.79% at a concentration of 200 μM for 6-OHDA. Pretreatment with GM1 and GM2 for 30 min significantly increased cell viability (Figure 3B). Meanwhile, after pretreatment with Madopar for 30 min, cell viability also significantly increased. Among these, GM2 demonstrated greater efficacy in resisting 6-OHDA-induced neurotoxicity than GM1. In summary, GM1, GM2, and Madopar inhibited cell death induced by 6-OHDA in SH-SY5Y cells, demonstrating significant neuroprotective effects. Findings were consistent with our previous studies and verified the successful preparation of GMn.

### 2.3. GM2 Improved Motor Behavior in MPTP-Induced PD Mice

**Figure 4:**
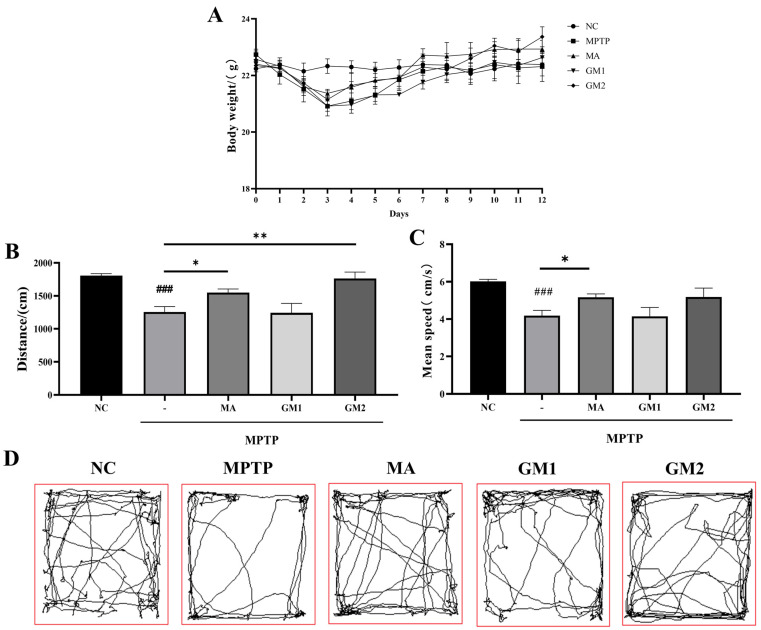
GM2 improved movement behavioral deficits in PD mice. (**A**) Body-weight growth curve of mice. (**B**) Total distance traveled in open field test. (**C**) Average speed of open field test. (**D**) Trajectory diagram of open field test. NC: control group; MPTP: mice treated with 30 mg·kg^−1^ MPTP alone; MA: mice treated with MA (70 mg·kg^−1^) followed by MPTP. GM1 and GM2: mice treated with 20 mg·kg^−1^. The values are given as mean ± SEM. ^###^ *p* < 0.001 versus control group; * *p* < 0.05 or ** *p* < 0.01 versus MPTP group.

The mice were observed and weighed daily throughout the experiment. Following MPTP treatment, the mice exhibited typical PD behaviors, such as reduced locomotion, bowed back, anorexia, and weight loss (Figure 4A). However, these symptoms were partially alleviated by GM2 treatment (i.g.). The open field experiment is a valid and convenient method to evaluate the movement behavior of PD mice. As shown in Figure 4B,C, the results revealed that MPTP significantly reduced the total traveled distance and mean speed of PD mice. Conversely, oral administration of GM2 effectively improved the movement distance of PD mice (Figure 4B) but did not significantly increase the mean speed (Figure 4C). These results are consistent with those shown in Figure 4D (activity trajectory graph). However, GM1 treatment at the same dose showed no significant changes. These results demonstrated that the oral administration of GM2 had a neuroprotective effect on PD mice. The MA group, by directly supplying the precursor of dopamine–levodopa, exhibited enhanced motor abilities. In comparison with the MPTP group, the MA group demonstrated significantly improved motor capabilities, consistent with the findings of Pan et al. [26].

### 2.4. GMn Attenuated Dopamine Neuronal Damage in MPTP-Induced PD Mice

**Figure 5:**
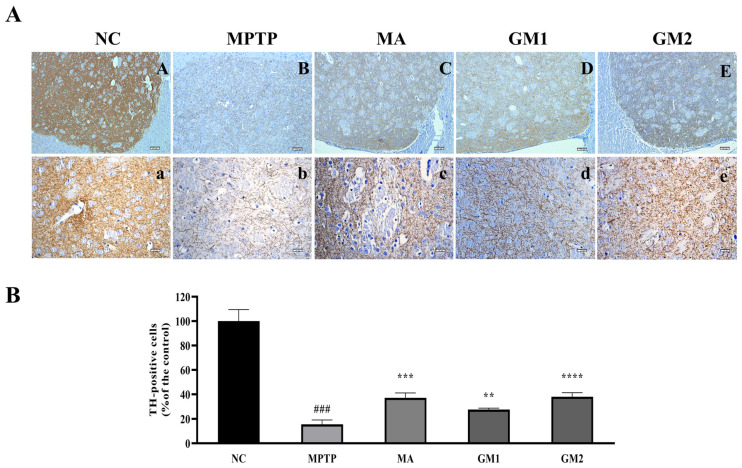
GMn alleviates MPTP-induced loss of dopamine neurons in PD mice. (**A**) Representative micrographs of immunohistochemical staining of TH in the striatum. Scale bars (A–E) 100 μm, (a–e) 20 μm. (**B**) Quantification of TH-positive cells in the striatum for each group. The values are given as mean ± SEM. ^###^ *p* < 0.001 versus control group; ** *p* < 0.01 or *** *p* < 0.001 or **** *p* < 0.0001 versus MPTP group.

Tyrosine hydroxylase (TH) is a crucial enzyme in dopamine synthesis, and its decreased expression constitutes a primary pathological characteristic of PD [27]. In order to investigate the effects of GMn on TH expression, we assessed the expression levels of TH protein in the striatum using an immunohistochemistry (IHC) method. Compared to the control group, the number of TH-positive cells in the striatum of MPTP-induced PD mice was significantly reduced, while GM1 and GM2 treatments effectively reversed the loss of dopaminergic neurons in the striatum induced by MPTP (Figure 5). The study demonstrated that Madopar significantly mitigated the neurodegenerative alterations induced by MPTP in the striatum [28,29]. In this study, similar effects were also observed in the MA group.

### 2.5. GMn Maintained Homeostasis of Monoamine Neurotransmitters

PD is caused by a combination of environmental and genetic factors, leading to abnormalities in dopamine metabolism in the substantia nigra and a decrease in dopamine levels in the striatum, resulting in motor symptoms [30]. Therefore, we investigated the impact of GMn on the alterations of striatal neurotransmitters induced by MPTP by assessing the levels of DA and its metabolites in the striatum. In comparison to the control group, MPTP significantly reduced the levels of DA, DOPAC, and HVA in the striatum. However, GM1 effectively reversed the MPTP-induced abnormal elevation of the ratio of (DOPAC + HVA)/DA in the striatum, indicating that GM1 significantly inhibited the turnover of DA in the striatum (Table 4). In summary, GM1 and GM2 were found to prevent the decrease in dopamine metabolites, DOPAC and HVA, and partially maintain the homeostasis of monoamine neurotransmitters to some extent.

### 2.6. GMn Alleviated the Dysbiosis of Gut Microbiota in MPTP-Induced PD Model Mice

**Figure 6:**
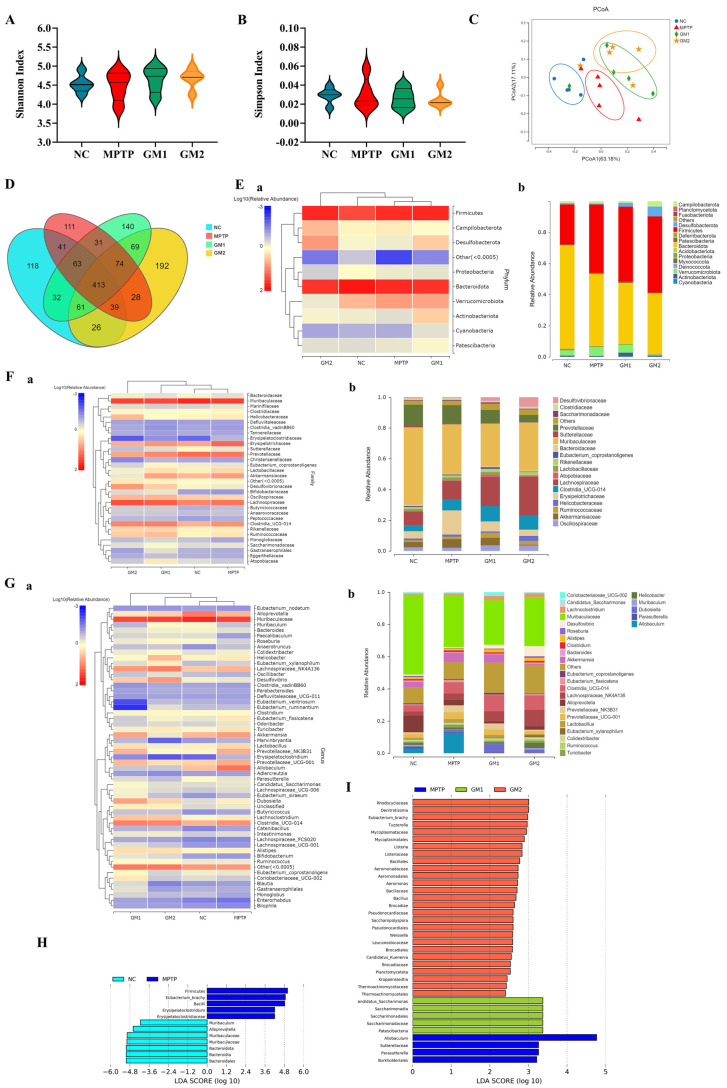
Effects of GMn on gut microbiota in MPTP-induced PD model mice. (**A**,**B**) Analysis of α-diversity: the Shannon and Simpson indexes. (**C**) PCoA of β-diversity. (**D**) Venn diagram. (**E**) Relative abundance of gut microbiota at the phylum level: (**a**) heat map: red, high expression; blue, low expression; (**b**) bar graph. (**F**) Relative abundance of gut microbiota at the family level: (**a**) heat map; (**b**) bar graph. (**G**) Relative abundance of gut microbiota at the genus level: (**a**) heat map; (**b**) bar graph. (**H**) Microbial structure and the predominant bacteria between the control group and MPTP group; linear discriminant analysis, LDA > 2. (**I**) Microbial structure and the predominant bacteria between the MPTP group and the GMn group, LDA > 2. The values are given as mean ± SEM.

To investigate the effect of GMn on gut microbial composition in MPTP-induced PD model mice, we used 16S rRNA sequencing and combined it with a bioinformatics analysis. Shannon’s index and Simpson’s index were used to evaluate changes in the α-diversity of the gut microbiota in mice, with higher values indicating greater diversity. The results indicated that there were no statistically significant differences in Shannon’s index and Simpson’s index between the control group, MPTP group, and GMn group (Figure 6A,B). Additionally, we evaluated β-diversity using a principal coordinate analysis (PcoA) based on weighted UniFrac distances to determine the similarity of the gut microbial community between groups. As shown in Figure 6C, while variations were observed within each group, the microbial communities of the MPTP group distinctly separated from the other three groups, with a higher similarity between GM1 and GM2 groups. These findings suggested that GMn exerted an influence on the intestinal microbiota composition in MPTP-induced PD mice. The Venn diagram analysis further revealed 413 common operational taxonomic units (OUTs) among the four groups with GMn groups exhibiting a greater number of OUTs compared to the MPTP group. Specifically, the GM1 group had 140 unique OUTs, and the GM2 group had 192 unique OUTs (Figure 6D). To investigate the abundance and distribution of gut microbiota, and potential flora associated with microbial ecological dysbiosis, we observed significant changes in microbiota composition at the phylum, family, and genus levels. At the phylum level, the predominant phyla were Bacteroidota and Firmicutes. The relative abundance of Firmicutes was elevated, while Bacteroidota decreased in the MPTP group compared to the control group (Figure 6E(a)). Meanwhile, the activity heat map of Figure 6E(b) illustrated that within the MPTP group, there was a decline in the relative abundance of Actinobacteria and Desulfotomaculum, whereas there was an increase in the relative abundance of Patescibacteria, Campilobacterota, and Verrucomicrobiota. Compared with the MPTP group, the alterations were effectively reversed following treatment with GMn. Notably, except for the increased relative abundance of Actinobacteriota and Desulfobacterota within the GM1 and GM2 groups, respectively, the relative abundance of Campilobacterota decreased in the GM1 group, while Verrucomicrobiota and Patescibacteria decreased in the GM2 group. The relative abundance of Firmicutes and Bacteroidota was minimally affected by GM1 and GM2, with no statistically significant differences. At the family level, the dominant microbiota changed with a decrease in the relative abundance of Helicobacteraceae and an increase in the relative abundance of Desulfovibrionaceae, Erysipelotrichaceae, and Eubacterium_coprostanoligenes after GM1 treatment compared to the MPTP group (Figure 6F). Similarly, after GM2 treatment, there was a decrease in the relative abundance of Lactobacillaceae and an increase in the relative abundance of Bacteroidaceae and Erysipelotrichaceae. At the genus level, in PD mice compared to the control group, MPTP induced a decrease in the relative abundance of *Bacteroides*, *Roseburia*, and *Desulfovibrio*, and an increase in the relative abundance of *Akkermansia*, *Lactobacillus*, and *Revotellaceae_UCG-001* (Figure 6G). However, in PD mice treated with GM2, the opposite result was found, as there were no significant changes in the GM1 group. The gut microbiota abundance in most of the GM2-treated PD mice was similar to that of the control mice. The study analyzed the microbial structure and dominant bacteria in each group using LEfSe LDA plots from the phylum to genus levels (Figure 6H,I). The results revealed that the control group enriched seven bacterial taxa, while the MPTP and GM1 groups enriched four and five bacterial taxa, respectively. Notably, the GM1 group showed an enrichment in 27 bacterial taxa. The findings demonstrated that the microbial composition was significantly altered in PD mice. GMn was found to modulate microbiota composition, especially GM2, and ameliorated MPTP-induced gut microbiota dysbiosis in PD model mice.

**Figure 7:**
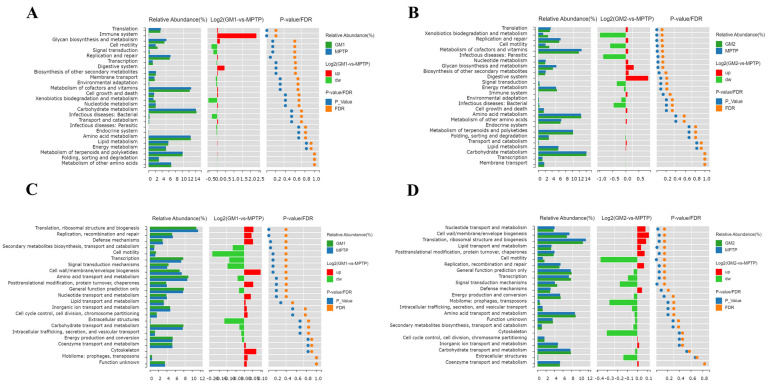
GMn can modify the expression of 16S functional genes and metabolic signaling pathways in the intestinal microbiota of a mouse model of PD. (**A**,**B**) KEGG functional prediction analysis of GMn. (**C**,**D**) Clusters of orthologous groups (COGs) of proteins’ functional prediction analysis of GMn.

To explain the functions of these altered bacteria, KEGG pathway and COGs analyses were performed. As illustrated in Figure 7A, compared to the MPTP group, GM1 downregulated metabolic pathways such as translation and the immune system. GM2 upregulated xenobiotic degradation, metabolism, and cell motility, while downregulating metabolic pathways such as auxiliary factors and vitamin metabolism, nucleotide metabolism, glycan biosynthesis and metabolism, as well as the translation, replication, and repair of genetic information (Figure 7B). The analysis of clusters of orthologous groups (COGs) of proteins revealed a significantly higher abundance of proteins related to signal transduction and cell motility in the GM2 group compared to the MPTP group. Conversely, the abundance of proteins associated with lipid transport and metabolism and nucleotide transport and metabolism were significantly lower in the GM2 group compared to the MPTP group (Figure 7D). No significant difference was found in the GM1 group (Figure 7C).

## 3. Discussion

Oligosaccharides, abundant and widely distributed biomolecules in nature, play a crucial role in various physiological processes. Our previous studies showed that GM2 was a glucuronomannan tetrasaccharide obtained from sulfated polysaccharides by a dilute acid hydrolysis and multistep chromatography. However, the current GMn preparation process has several drawbacks, making the preparation of GMn a challenging step. In this study, a two-factor, three-level orthogonal test was meticulously designed to optimize the preparation process of GMn. The initial material was SPF2, characterized by a high glucuronic acid and mannose content, obtained from the degradation of crude fucoidan from *S. japonica*. The degradation temperature and time of SPF2 were examined. The study found that the highest relative abundance of GMn in the degraded products was achieved under specific conditions: a final sulfuric acid concentration of 4%, a reaction temperature of 100 °C, and a 3.5 h heating reflux reaction. The percentages of GM1 and GM2 in the degradation products were 17.36% and 13.44%, respectively. After purification, the yield of GMn increased by 400~800 times compared to the previous preparation process, with a concurrent reduction in preparation time by one-third. The mild acid hydrolysis technique emerged as a simple, inexpensive, and convenient hydrolysis method for polysaccharides. However, its unspecific nature resulted in a range of oligosaccharides, complicating the subsequent purification. Research has demonstrated that prolonged hydrolysis times lead to a reduced molecular weight [31]. Ronge et al. discovered that using a 2 M HCl solution to hydrolyze chitosan resulted in a molecular weight of 200 kDa after 30 min and 17 kDa after 1 h [32]. Moreover, as the reaction time prolonged, the yield of hydrolysis products decreased, consistent with the findings of this study. Therefore, the refined process not only eliminated multiple steps of chromatography, streamlining the overall procedure, but also significantly enhanced the content of GMn in the degradation products while shortening preparation time. This advancement established a foundation for the subsequent in-depth research on GMn.

In patients with PD, gastrointestinal dysfunction, such as dysphagia, delayed gastric emptying, and constipation, may occur decades before the emergence of motor symptoms [18]. The gut microbiota play a crucial role in the complex interconnections between the gastrointestinal system and the brain, particularly in neurological and metabolic disorders. The gut microbiota produce neurotoxic metabolites that enter the central nervous system through the bidirectional gut–brain axis, thereby affecting brain function [22]. Thus, the modulation of the gut microbiome emerges as a novel therapeutic avenue for managing the onset and progression of PD. Oral administration is the primary route for delivering drugs into the gastrointestinal tract, providing a direct means of influencing the composition of gut microbiota. This approach is widely accepted and adhered to by patients, offering convenience and safety compared to alternative methods like injections or surgery, thereby circumventing associated discomfort and complications. This is especially crucial for the ongoing management of chronic conditions like PD. To investigate the neuroprotective mechanism of gavage-administered GMn against PD, we first verified its protective effect against 6-OHDA-induced cell death in vitro using SH-SY5Y cells. Our study revealed that pretreatment with GM1 or GM2 significantly inhibited 6-OHDA induced SH-SY5Y cell death Thus, GMn demonstrated significant neuroprotective effects in an in vitro cell model.

We established a subacute animal model of PD induced by MPTP to elucidate the potential mechanism of action of orally administered GMn. Following the induction of PD with MPTP at a dose of 30 mg/kg/day for 5 consecutive days, the mice exhibited significant motor behavioral deficits, decreased levels of DA, 5-HT, and its metabolites in the striatum, and a loss of TH-positive neurons, indicating a successful PD model. It has been reported that different modes of administration lead to distinct mechanisms of action, resulting in varying research outcomes [33]. Notably, only oral administration of GM2 (100 mg/kg) for 7 days improved the activity capacity of PD mice in this study. Furthermore, oral administration of GM1 and GM2 did not significantly alter the content of DA but markedly inhibited the decrease in MPTP-induced DA metabolites, DOPAC, and HVA, which was partially inconsistent with the results of previous studies involving an injected administration of GMn. This suggested that the anti-PD effects of oral administration of GM2 were exerted through mechanisms distinct from those of injected administration [16]. Immunohistochemical staining results demonstrated that both GM1 and GM2 effectively mitigated the loss of dopaminergic neurons in the striatum. Additionally, in this study, Madopar (70 mg/kg) showed effects similar to GM1/2 (100 mg/kg), establishing Madopar as the “gold standard” drug for PD treatment. These findings indicated that GM2 had the potential to ameliorate behavioral deficits, protected the dopaminergic pathway, and prevented the loss of dopaminergic neurons in PD animals, whether administered orally or by injection.

Studies have shown that both PD patients and MPTP-induced PD mice exhibit not only dysbiosis in the gut microbiota but also significant differences in the abundance and composition of intestinal microorganisms [34]. Our analysis, based on β-diversity and species composition, indicated significant differences in the relative abundance and distribution of gut microbiota at the phylum, family, and genus levels among the control, MPTP, GM1, and GM2 groups of mice, which was consistent with findings by Wu et al. [35]. Furthermore, the experimental results indicated that gut microbiota’s ecological dysbiosis in PD mice was primarily characterized by a decrease in the relative abundance of *Prevotellacea* and *Roseburia*, coupled with an increase in the relative abundance of the Verrucomicrobiota phylum and its family, Akkermansiaceae. Clinical studies have observed significant changes in the β-diversity of the gut flora in PD patients [36], with a significant increase in Lactobacillaceae and Verrucomicrobiales and a significant decrease in Larabacteriaceae and *Prevotella* [37]. Furthermore, the duration of PD has shown an inverse correlation with the presence of short-chain fatty acid-producing bacteria and probiotics but a direct correlation with pathogenic bacteria [38]. Meanwhile, Zhang et al. discovered that the abundance of *Akkermansia* and Verrucomicrobiota significantly increased in PD patients, while *Prevotellacea* decreased [39], aligning with our study’s findings. Studies have shown that the increased degradation of mucin is associated with a higher abundance of *Akkermansia* in patients with PD and AD [40,41]. *Akkermansia muciniphila* had the capability to degrade mucins within the intestinal mucosal layer, inducing an increase in intestinal permeability [41,42]. In this study, GM2 treatment reduced the relative abundance of Akkermansiaceae, which belongs to Verrucomicrobiota in PD mice, suggesting GM2 may protect the mucus layer by inhibiting the degradation of mucin. Consequently, this protected the enteric neurons, inhibiting the onset and progression of PD. Conversely, GM1 showed no significant changes.

Additionally, both GM1 and GM2 inhibited the abnormal reduction in *Prevotellaceae_NK3B31* and *Prevotellaceae_UCG-001* abundance (Figure 6G). Studies have shown that a decrease in *Prevotella* abundance is associated with lower levels of the gut growth hormone-releasing peptide [43]. This hormone affects the function of dopaminergic neurons in the substantia nigra compacta and is involved in the development of degenerative neurological disorders [44]. Additionally, the abundance of *Prevotella* significantly decreases with PD progression [45]. Thus, the decrease in *Prevotella* levels serves as a highly specific biomarker for PD. *Roseburia*, a bacterium belonging to the Firmicutes phylum, is recognized for its capacity to generate butyrate. Liu et al. observed a decreased abundance of *Roseburia* and a significant reduction in fecal butyrate levels in individuals with PD [46]. Moreover, a negative correlation was identified between the severity of PD and the abundance of *Roseburia*. Butyrate salts supported intestinal barrier function by stimulating the production of tight junction components and mucin. Additionally, they elicited various physiological responses through G protein-coupled receptors (GPCRs) in intestinal cells [47]. A study demonstrated that in a PD mouse model, oral administration of sodium butyrate increased colonic GLP-1 levels, upregulated GLP-1 receptors (GLP-1R) in the brain, and improved neurobehavioral disorders [48]. GM2 treatment increased the abundance of *Roseburia* in PD mice, potentially by restoring levels of butyrate in the intestinal tract. This enhancement contributed to the stabilization of the intestinal barrier and the stimulation of gut hormone secretion. Consequently, these effects may lead to an amelioration of MPTP-induced behavioral deficits, suggesting a beneficial effects on PD mice.

Although *Lactobacillus* was known as a probiotic with anti-inflammatory effects, our study revealed a higher abundance of *Lactobacillus* and its affiliation with the Lactobacillaceae family in PD mice, which was consistent with findings in PD patients [49]. This suggests that the role of *Lactobacillus* in PD may be more complex than previously assumed. Li et al. observed a significant correlation between clinical indicators of inflammation, such as the percentage of neutrophil granulocytes, monocytes, and monocyte count, and an increase in Bifidobacteriaceae, *Bifidobacteria*, Lactobacillaceae, and *Lactobacillus* [50]. GM1 and GM2 were effective in reducing *Lactobacillus* levels. Further studies are needed to investigate the relationship between *Lactobacillus*, GM1, GM2, and PD progression. Therefore, GM1 and GM2 may exert neuroprotective effects against PD by alleviating MPTP-induced gut microbiota dysbiosis, improving enterocolitis, restoring intestinal barrier integrity, and ameliorating disorders of short-chain fatty acid metabolism. The neuroprotective effect of GM2 was found to be more significant compared to GM1, consistent with prior research findings [16].

## 4. Materials and Methods

### 4.1. Chemicals and Reagents

Madopar (MA) was purchased from Shanghai Roche Pharmaceuticals Co., Ltd. (Shanghai, China). 6-OHDA was purchased from Aladdin (Shanghai, China). MPTP was purchased from Bide Pharmatech Ltd. (Shanghai, China). Dopamine (DA), 3,4-dihydroxyphenylacetic acid (DOPAC), homovanillic acid (HVA), 5-hydroxytryptamine (5-HT), and 5-hydroxyindoleacetic acid (HIAA) were purchased from Sigma-Aldrich (St. Louis, MO, USA), and BCA protein assay kits were purchased from Beyotime Biotechnology (Nanjing, China). The PVDF transfer membrane was purchased from Millipore (Bedford, MA, USA). TH and β-actin antibodies were obtained from Santa Cruz Biotechnology (Santa Cruz, CA, USA).

### 4.2. Preparation of Glucuronomannan Oligosaccharides (GMn)

GMn were prepared following an optimization of the scheme reported in a previous study (Figure 1A) [15]. Briefly, to optimize the separation route, the sulfated heteropolysaccharide (SPF2), derived from the degradation of crude fucoidan obtained from *Saccharina japonica* [51], was used for subsequent degradation. SPF2 was further degraded using the 4% dilute acid. The resulting solution was neutralized with barium hydroxide and centrifuged. The supernatant was then absorbed by activated carbon (Sinopharm Holdings Chemical Reagent Co., AR, Shanghai, China). Elution was carried out with ethanol at different concentrations, yielding two fractions: the aqueous-eluted fraction (Y1) and the 50% ethanol-eluted fraction (Y2). Fraction Y2 was purified through a Bio-Gel P-4 gel column (Extra Fine, <45 μM; 2.6 cm × 100 cm) (GE, Uppsala, Sweden) and eluted with 0.5 M NH_4_HCO_3_ solution. The optimized preparation process described above is illustrated in Figure 1B. The elution results were determined using the phenol–sulfuric acid method. The six major fractions were collected, concentrated, and lyophilized.

### 4.3. Identification and Purity of GMn

The relative abundance of all fractions was determined by ESI-MS in the negative ion mode at the *m*/*z* range from 200 to 2000.

NMR was employed to validate the structural features of GMn (Figure S1). GMn (7 mg) were dissolved in deuterated oxide (99.9%) and subjected to two cycles of consecutive freeze-drying, followed by dissolution in deuterated oxide (99.9%). One-dimensional NMR spectra were acquired using a Bruker AVANCE III instrument (Bruker BioSpin, Billerica, MA, USA) operating at 600 MHz and 25 °C.

The purity of GMn was analyzed on an HPLC (Shimadzu, Japan) equipped with an ELSD detector (Alltech 2000ES, Alltech Associates, Inc., Nicholasville, KY, USA) using a Click Xlon column (Acchrom^TM^, 5 μm, 4.6 × 250 mm). The dried test sample was dissolved in distilled water (5 mg/mL). The column oven temperature was set at 30 °C, and the mobile phase buffer was a 100 mmol/L formic acid–ammonium formate solution (pH 3.2), with a gradient elution at a flow rate of 1 mL/min. The gradient ranged from 65:35 to 40:60 acetonitrile–buffer solution over 0–30 min. Each sample was equilibrated with the initial mobile phase for 10 min before testing. The ELSD detector parameters were set as follows: evaporation temperature of 85.7 °C and gas flow rate of 2.2 L/min.

### 4.4. Optimization of Degradation Conditions

To increase the GMn yield, an orthogonal experiment was conducted to optimize the SPF2 degradation process. The effects of degradation temperature (A) and degradation time (B) on GMn yield in the degradation products were investigated using the orthogonal design. Table 5 shows the experimental factor levels.

### 4.5. Cell Culture and Treatment

SH-SY5Y cells were used to assess the neuroprotective effect of GMn and their effects on the expression of tyrosine hydroxylase, which are commonly used as cellular models for PD. The cells were cultured in an incubator at 37 °C with 5% CO_2_ concentration using a DMEM/F12 medium supplemented with 10% fetal bovine serum (Gibco), 100 μg/mL penicillin, and streptomycin. For all experiments, cells were seeded in 96-well plates at a density ranging from 1 × 10 ^4^ to 1 × 10 ^5^ cells/mL.

Cells were treated with varying concentrations of 6-OHDA for 24 h to assess cytotoxicity. Prior to exposure, cells were pretreated with GM1 (200 μg/mL) or GM2 (200 μg/mL) for 30 min, followed by incubation with 6-OHDA for 24 h. Control cells were treated with an equivalent volume of PBS and 100 mM MA was used as a positive control.

### 4.6. Cell Viability Assay

Cell viability was assessed through the MTT assay [52]. Briefly, 10 μL of MTT solution (5 mg/mL) was added to each well following cell treatment and incubated for 4 h. After removing the medium, 150 μL of DMSO solution was added to each well. The culture plate was then placed on a microplate oscillator and shaken for 10–15 min to dissolve the crystals with DMSO. Finally, the absorbance at 490 nm was measured using a Multiskan EX microplate reader (Thermo Fisher Scientific, Waltham, MS, USA). The cellular viability percentage was calculated using the following formula: cell viability (%) = treated cells/control cells × 100.

### 4.7. Animals and Treatment

Male C57BL/6 mice, aged 6–7 weeks and weighing 20 ± 2 g, were purchased from Jinan Pengyue Laboratory Animal Center. The study was approved by the Institutional Animal Care and Use Committee (IACUC) of Qingdao University, and it adhered to the principles of laboratory animal care established by the National Society for Medical Research. The laboratory animal license number was SCXK (Lu) 2022 0006, with approval granted on 12 May 2023. The mice were housed in an SF-grade animal facility with free access to food and water, a 12/12 light and dark cycle, 50–55% humidity, and a temperature range of 22–24 °C. Prior to the experiment, the mice were acclimatized and fed for five days.

**Figure 8:**
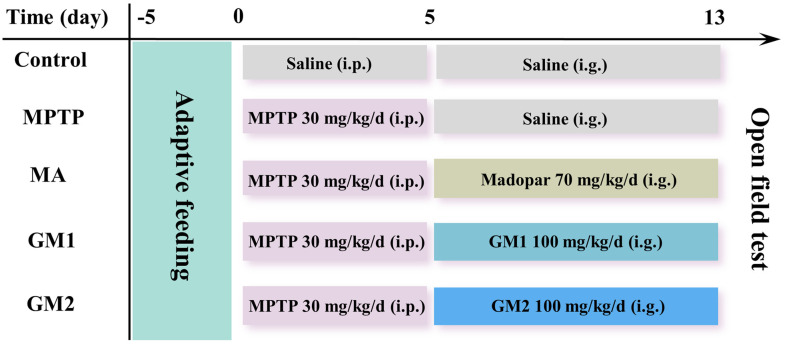
Schematic diagram of the experimental procedure.

The mice were randomly assigned to five groups (*n* = 6 mice/group): control (NC), MPTP, MPTP + MA (70 mg/kg), MPTP + GM1 (100 mg/kg), and MPTP + GM2 (100 mg/kg). To induce experimental PD syndrome, mice were injected intraperitoneally with MPTP (30 mg/kg, dissolved in sterile saline) once daily for 5 consecutive days. The control group received an equivalent volume of saline. Afterwards, the GM1, GM2, and MA groups were administered GM1, GM2, or MA by oral gavage for 7 days, while the control and MPTP groups received the same volume of saline. Two hours after the final oral administration, four mice from each group were randomly selected for open field experiments to assess behavior and movement. At the end of the experiments, three mice from each group were anesthetized and perfused via intracardial infusion with 0.9% saline, followed by 4% paraformaldehyde. The whole brains were removed and paraffin-embedded to prepare serial coronal sections. In each group, striatal tissues from three additional animals were rapidly dissected on ice and stored at −80 °C until use. The experimental design is illustrated in Figure 8.

### 4.8. Open Field Test

Before conducting the experiment, mice were allowed to acclimate to the environment. During the 5 min experimental session, each mouse was placed in the center of a square box (50 × 50 × 30 cm) with a white floor, and the camera positioned at the top center recorded the mouse’s movement trajectory. The trajectories, total distance traveled (cm), and average speed (cm/s) during the observation period were analyzed and recorded using animal tracking software (SMART 3.0). The experimental area was cleaned with 75% alcohol before each test to eliminate any potential biases caused by the scent of the previous mouse [53].

### 4.9. Immunohistochemistry

To evaluate neuronal loss in the striatum of mice with PD, we performed tyrosine hydroxylase (TH) staining. Following behavioral testing, mice were anesthetized with chloral hydrate, perfused with saline and 4% paraformaldehyde (PFA). The brains were subsequently removed and fixed in a 4% paraformaldehyde solution before embedding the brain tissue in paraffin. The striatal tissues were consecutively sliced with a thickness of 5 μm using a Leica microtome (RM2235; Leica Microsystems GmbH, Wetzlar, Germany) and placed on polylysine-treated slides. The paraffin sections underwent dewaxing, hydration, and high-temperature antigen repair using a citrate antigen repair buffer (pH 6.0). To minimize nonspecific background staining, sections were incubated with a 3% hydrogen peroxide solution for 10 min at room temperature to block endogenous peroxidase activity. The slides were washed three times in PBS (pH 7.4) for 3 min each. Subsequently, the sections were incubated overnight with TH antibody (1:500 sc-25269) at 4 °C overnight. After three washes with PBS (pH 7.4) for 3 min each, the sections were incubated with a horseradish peroxidase-coupled anti-mouse IgG secondary antibody (1:500, GB23301) at room temperature for 2 h. Following this, the sections underwent 3,3′-diaminobenzidine (DAB) color development and hematoxylin re-staining. Finally, they were dehydrated transparently with ethanol and xylene and sealed with neutral gum. Positive antibody expression in the sections appeared as deep brown. Images were captured using a microscope (BX41, Olympus Corp, Tokyo, Japan). Quantitative analysis was performed using Image-Pro Plus 6.0 (Media Cybernetics, Silver Spring, MD, USA) through the density measurement method.

### 4.10. HPLC-EDC Analysis

The levels of DA, DOPAC, HVA, 5-HT, and 5-HIAA were measured using an HPLC-EDC assay system (Thermo Fisher Scientific, Waltham, MA, USA). Briefly, the striatal tissues, which were stored at −80 °C, were freeze-homogenized in a solution containing 0.1 M HClO_4_, 0.1 mM EDTA-2Na, and 4 × 10^−8^ M internal standard DHBA. The samples were centrifuged at 4 °C, 20,000 rpm for 30 min. The supernatant was filtered through a 0.22 μm Millipore filter and then injected into an HPLC system for analysis. Separation was achieved using a Thermo Acclaim^®^ separation C18 column (2.1 × 100 mm, 2.2 μm). Mobile phase: 90 mM NaH_2_PO_4_, 50 mM citric acid, 1.7 mM OSA, 50 μM EDTA, 4.3% acetonitrile. The voltage of the detection cell was 350 mv, the voltage of the guard cell was 360 mv, the response range was 100 nA, and the flow rate was 0.2 mL/min. The data were collected and analyzed using the Chromeleon 6.9 chromatography workstation, and the target concentration was calculated using the internal standard method.

### 4.11. 16S rRNA Gene Sequencing

The influence of GM1 and GM2 on PD-induced changes in microbial communities was assessed through the sequencing of gut microbes. Fresh fecal samples were collected from mice under sterile conditions on day 7 after drugs’ administration and stored at −80 °C until further use. Total DNA was extracted and purified from fecal contents using the KingFisher fully automated nucleic acid extraction system (Thermo Fisher Scientific, Waltham, MA, USA). The V3-V4 region of the 16S rRNA gene was amplified using DNA extracted from each sample as a template, followed by the purification of PCR amplicons using Agencourt AMPure XP magnetic beads and elution in an elution buffer, labeling, and library construction. The fragment range and concentration of libraries were assessed using the Agilent 2100 Bioanalyzer, and qualified libraries were sequenced on the Illumina MiSeq platform based on the size of the inserted fragments. The raw sequencing data underwent quality filtering to obtain clean data [54], followed by assembly using FLASH [55]. Operational Taxonomic Units (OTUs) were generated by clustering sequences at a 97% similarity threshold using UPARSE software (version 7.1). OTU representative sequences were annotated to species level against a reference database using RDP classifier software (version 1.9.1), with a confidence threshold set at 0.6. A beta diversity analysis of microbial communities was performed using QIIME [56] (v1.80), while the alpha diversity of gut microbiota was analyzed using QIIME (v.1.31.2). The functional prediction of microbial communities was conducted using PICRUST2 (v2.3.0-b). Metagenome sequencing and subsequent analyses were performed by BGI Genomics Co., Ltd. (Guangzhou, China).

### 4.12. Statistical Analysis

The data were expressed as mean ± SEM for three independent experiments. To compare differences between groups, a one-way analysis of variance (ANOVA) was used, followed by least significant difference (LSD) multiple comparison tests. All statistical analyses were performed using GraphPad Prism software (Version 9.4; La Jolla, CA, USA). Statistical significance was considered when *p* < 0.05.

## 5. Conclusions

This study optimized the degradation parameters of SPF2 and increased the yields of GM1 and GM2 in the degraded products. The optimal conditions for SPF2 degradation were achieved with a final sulfuric acid concentration of 4%, reaction temperature of 100 °C, and degradation time of 3.5 h. The degraded products reached the highest relative abundance of GMn under these conditions, with GM1 at 17.36% and GM2 at 13.44%, and the yield of GMn increased by 400~800 times compared to the previous preparation process. Oral administration of GM1 and GM2 modulated the composition of the gut microbiota in MPTP-induced PD mice. Moreover, GM2 effectively ameliorated behavioral deficits. Therefore, integrating findings from previous studies, both oral and injection routes demonstrated a significant neuroprotective activity of GM2, with GM2 exhibiting more pronounced effects compared to GM1. These findings reveal the potent role of GM2 in the treatment of PD, thereby advancing the exploration and development of GM2 as a potential therapeutic agent for Parkinson’s disease.

## Figures and Tables

**Table 1:** Relative abundance of GMn under the experimental conditions using an orthogonal array design.

The NO.	A-Hydrolysis Temperature	B-Hydrolysis Time	GM1 Relative Abundance (%)	GM2 Relative Abundance (%)
1	1	1	1.27	0.89
2	1	2	2.23	1.4
3	1	3	1.78	1.17
4	2	1	3.62	2.97
5	2	2	12.36	8.85
6	2	3	7.83	6.14
7	3	1	17.36	13.44
8	3	2	5.37	4.15
9	3	3	7.78	6.08

**Table 2:** Analysis of orthogonal design results.

	GM1 Relative Abundance (%)		GM2 Relative Abundance (%)	
	A-Hydrolysis Temperature	B-Hydrolysis Time	A-Hydrolysis Temperature	B-Hydrolysis Time
K1	5.28	22.25	3.46	17.30
K2	23.81	19.96	17.96	14.40
K3	30.51	17.39	23.67	13.39
k1	1.76	7.42	1.15	5.77
k2	7.94	6.65	5.99	4.80
k3	10.17	5.80	7.89	4.46
R	8.41	1.62	6.74	1.30

**Table 3:** ANOVA of two parameters for the relative abundance of GMn.

Source	Sum of Squares	DF	Mean Square	F Value	*p* Value	Significance
GM1						
A	113.867	2	56.934	3.954	0.064	ns
B	3.941	2	1.970	0.137	0.874	ns
Error	115.180	8	14.397			
GM2						
A	72.366	2	36.183	4.611	0.047	*
B	2.746	2	1.373	0.175	0.843	ns
Error	62.773	8	7.847			

“*” means that the factor is significant, “ns” means that the factor is not significant.

**Table 4:** DA, 5-HT, and their metabolites in the striatum (μg/g).

Groups	DA	DOPAC	HVA	5-HIAA	5-HT	(DOPAC + HVA)/DA
NC	0.511 ± 0.224	0.795 ± 0.116	0.983 ± 0.131	0.686 ± 0.101	0.042 ± 0.014	4.82
MPTP	0.161 ± 0.035 ^#^	0.294 ± 0.029 ^#^	0.701 ± 0.075 ^#^	0.586 ± 0.044	0.019 ± 0.001	7.55
GM1	0.417 ± 0.065	0.579 ± 0.042 *	0.971 ± 0.076 *	0.560 ± 0.033	0.027 ± 0.007	3.84 *
GM2	0.323 ± 0.101	0.589 ± 0.060 *	1.103 ± 0.052 *	0.687 ± 0.015	0.024 ± 0.003	5.31

The values are given as mean ± SEM. ^#^ *p* < 0.05 versus control group; * *p* < 0.05 versus MPTP group.

**Table 5:** Factors and levels for SPF2 degradation.

Factors	Levels		
	1	2	3
A-hydrolysis temperature (°C)	90	95	100
B-hydrolysis time (h)	3.5	4	4.5

## Data Availability

Data are contained within the article or Supplementary Materials.

## Supplementary Materials

The following are available online at https://www.mdpi.com/article/10.3390/md22050193/s1, Figure S1: 1H-NMR spectra of GMn. (A) 1H-NMR spectra of G2 (GM1) (B) 1H-NMR spectra of G3 (GM2).
