# Supplementary material for: Optimization of the Preparation Process of Glucuronomannan Oligosaccharides and Their Effects on the Gut Microbiota in MPTP-Induced PD Model Mice
Source: Mar Drugs. 2024 Apr 25;22(5):193. doi: 10.3390/md22050193 (PMC11123026; doi:10.3390/md22050193)
Supplement: Supplementary file 1 [file marinedrugs-22-00193-s001.zip › marinedrugs-2943095-supplementary.pdf]

Supplemental Material

Optimization of the preparation process of glucuronomannan oligosaccharides and their effects on the gut microbiota in MPTP-induced PD model mice

Baoxiang Wang[1,2], Lihua Geng[2,3,4,*], Jing Wang[2,3,4], Yuxi Wei[1], Changhui Yan [2,5], Ning Wu[2,4], Yang Yue[2,3,4], Quanbin Zhang[2,3,4,*]

1 College of Life Sciences, Qingdao University, 308 Ningxia Road, Qingdao 266003, China; wangbaoxiangcn@163.com; yuxiw729@163.com
2 CAS and Shandong Province Key Laboratory of Experimental Marine Biology, Institute of Oceanology, Chinese Academy of Sciences, Qingdao 266071, China; jingwang@qdio.ac.cn (J.W.); ych2747141850@163.com (C.Y.); wuning@qdio.ac.cn (N.W.); yueyang@qdio.ac.cn (Y.Y.)
3 Laboratory for Marine Biology and Biotechnology, Qingdao Marine Science and Technology Center, Qingdao 266237, China
4 Center for Ocean Mega-Science, Chinese Academy of Sciences, Qingdao 266071, China
5 Key Laboratory of Optic-electric Sensing and Analytical Chemistry for Life Science, MOE,College of Chemistry and Molecular Engineering, Qingdao University of Science and Technology, Qingdao 266042, PR China; ych2747141850@163.com
* Correspondence: lhgeng@qdio.ac.cn, Tel: +86-532-8289-8703; qbzhang@qdio.ac.cn, Tel: +86-532-8289-8708.

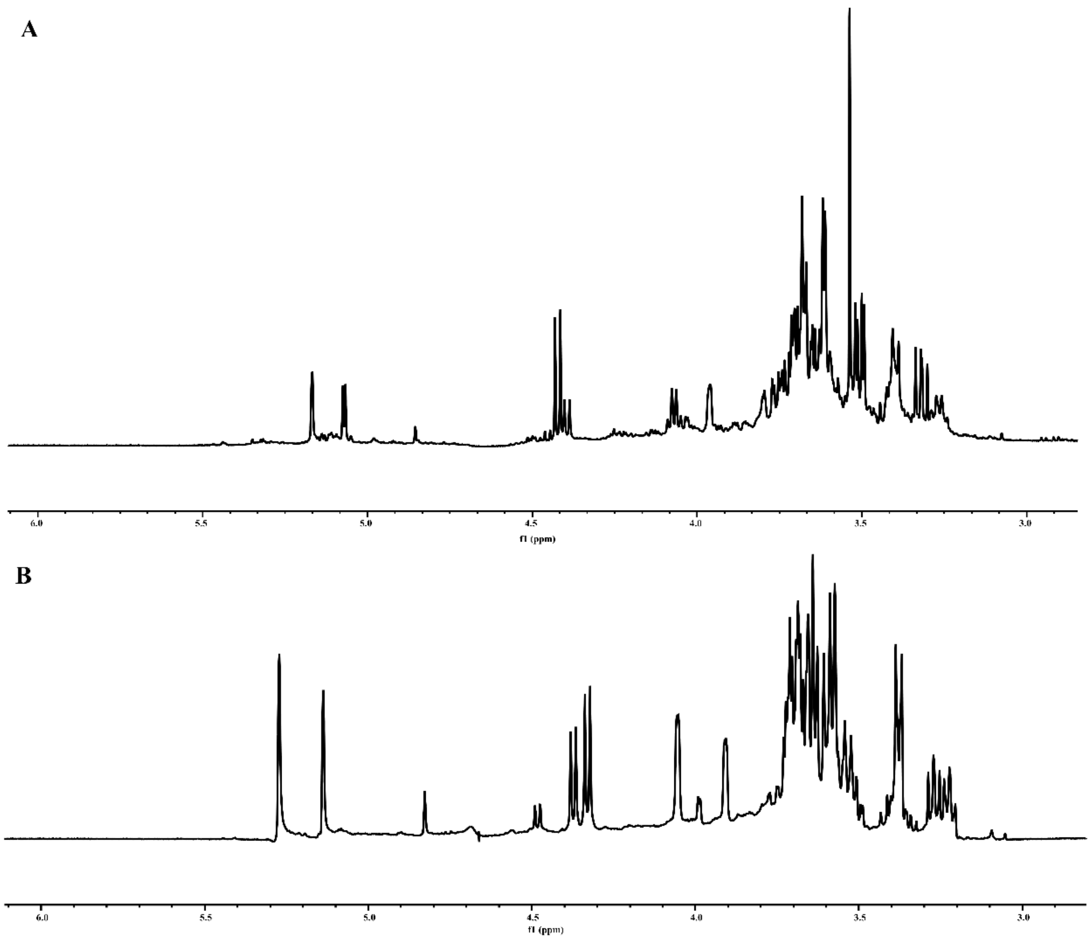

Figure S1. ^{1}H-NMR spectra of GMn. （A）^{1}H-NMR spectra of G2 (GM1) （B）^{1}H-NMR spectra of G3 (GM2).
